# Stent-Assisted Coiling in a Nickel-Allergic Patient

**DOI:** 10.7759/cureus.83055

**Published:** 2025-04-27

**Authors:** Shoichiro Tsuji, Yoji Kuramoto, Shinichi Yoshimura

**Affiliations:** 1 Neurological Surgery, Hyogo Medical University, Nishinomiya City, JPN

**Keywords:** coil embolization, complication of treatment, endovascular stenting, intracranial aneurysm, nickel allergy

## Abstract

We report the case of a woman in her fourth decade of life with a known nickel allergy. She visited the previous clinic complaining of a headache. Head MRI was performed, which showed a right internal carotid artery aneurysm, and she was referred to our hospital for further treatment. We planned treatment using coiling embolization with a balloon catheter because we were afraid of allergic reactions against the nickel device. However, recused stenting was done during the procedure to prevent coil migration. After the treatment, there were no post-procedure neurological complications or hypersensitivity reactions due to nickel allergy. No medication was given to suppress the hypersensitivity reaction. The patient was discharged from the hospital with no complications. This case demonstrates that nickel-containing stents can be safely used in selected patients with confirmed nickel allergy.

## Introduction

Endovascular treatment of intracranial aneurysms has spread rapidly in the last 20 years, with typical treatment devices such as coils and Woven EndoBridge devices implanted in the aneurysm and stents such as vascular reconstruction devices (VRDs) to prevent coil deviation and flow diverters (FDs), which are made from a variety of metals. Therefore, metal allergy is one of the obstacles to the endovascular treatment. Nickel is one of the most common allergens for metal allergies and is used in stents such as VRDs and FDs, which are used intracranially to maintain strength with shape memory and flexibility. Patients with metal allergies should be warned to avoid using instruments containing the offending metal in their treatment. However, if any troubles happen during the endovascular procedure, we have no choice but to use devices containing the metal to avoid serious complications, even for patients with metal allergies. We report our experience with a patient with a metal allergy who underwent coil embolization and was assisted with a metal stent to avoid complications. We report this case because we believe that sharing this case may be helpful in the future for treating aneurysms in patients with metal allergy, especially nickel allergy.

## Case presentation

**Figure 1:**
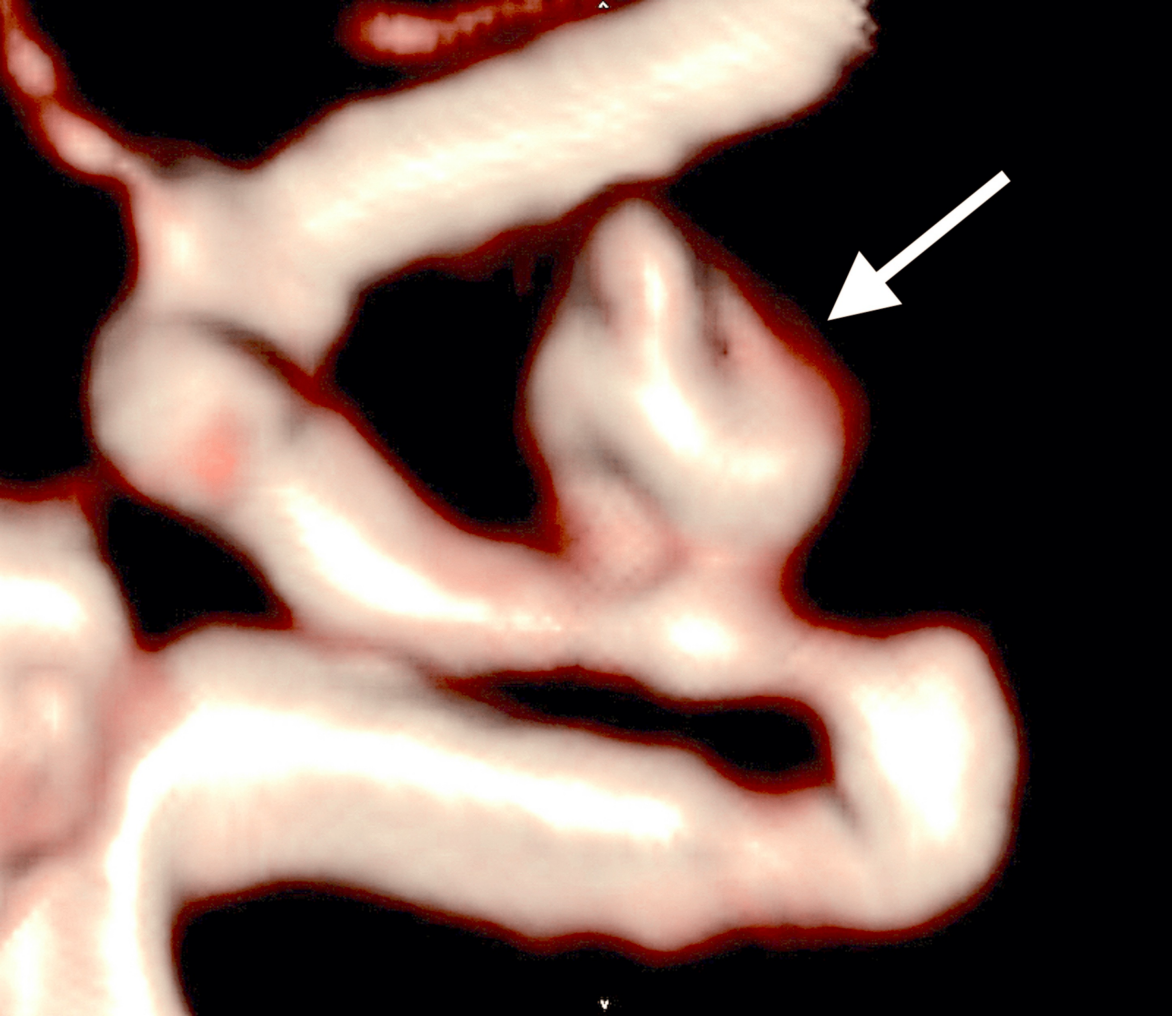
Initial MRA at previous hospital Oblique view of initial MRA showing a left paraclinoid aneurysm (white arrow). MRA, magnetic resonance angiography

**Figure 2:**
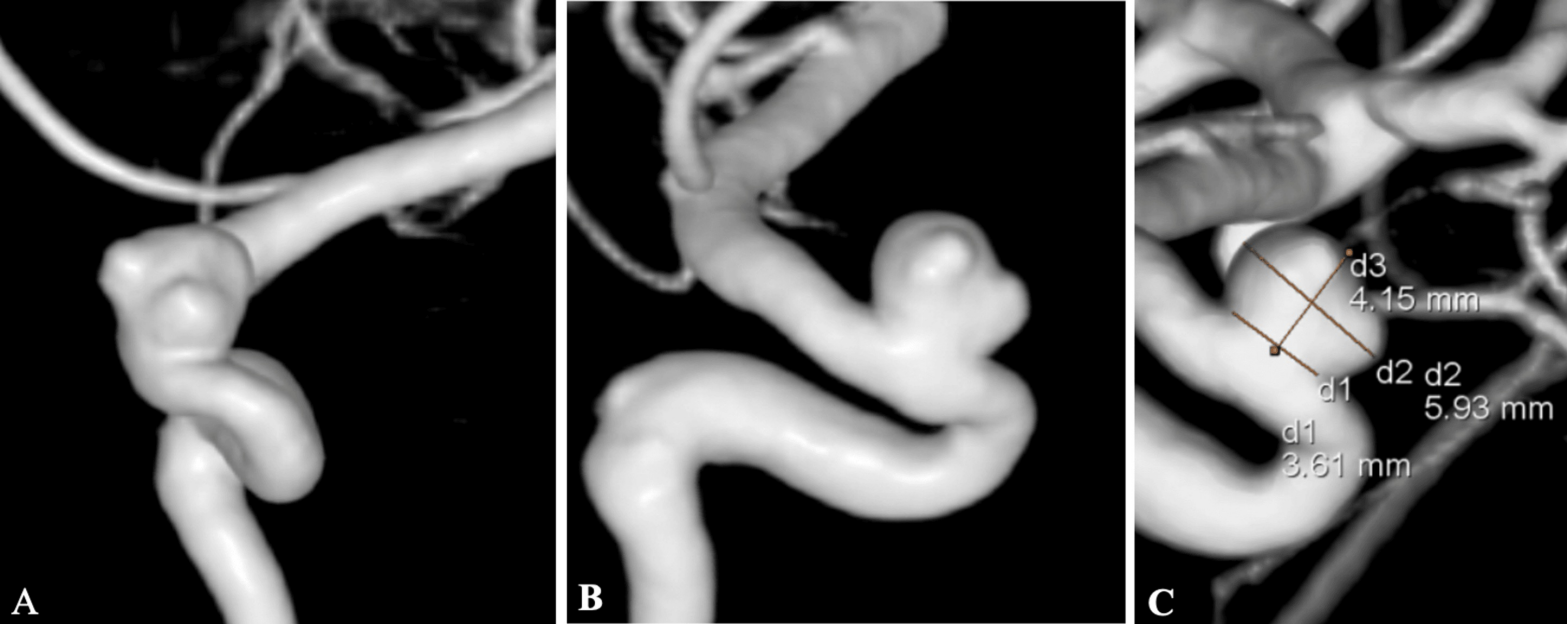
Pre-treatment 3D-RAs. (a) Frontal view. (b) Lateral view. (c) The size of the aneurysm. The neck was 3.61mm, the dome was 5.93mm, and the height was 4.15mm. 3D-RA, three-dimensional rotation angiogram

A female patient in her 40s visited a local clinic complaining of a headache. There was no identifiable cause of headache on MRI. However, a left paraclinoid aneurysm was incidentally found (Figure 1), and she was referred to our hospital for treatment. She had a past medical history of allergic rhinitis and hyperlipidemia. The aneurysm was located at the left internal carotid artery (ICA)’s anterior side of the supraclinoid portion. It had blebs; the maximum size was 5.62mm, the neck was 4.55mm, and the dome neck ratio was 1.4 (Figure 2).

The left ophthalmic artery did not originate from the left ICA but from the middle meningeal artery (MMA), and this might be the cause of blindness during craniotomy surgery.

**Figure 3:**
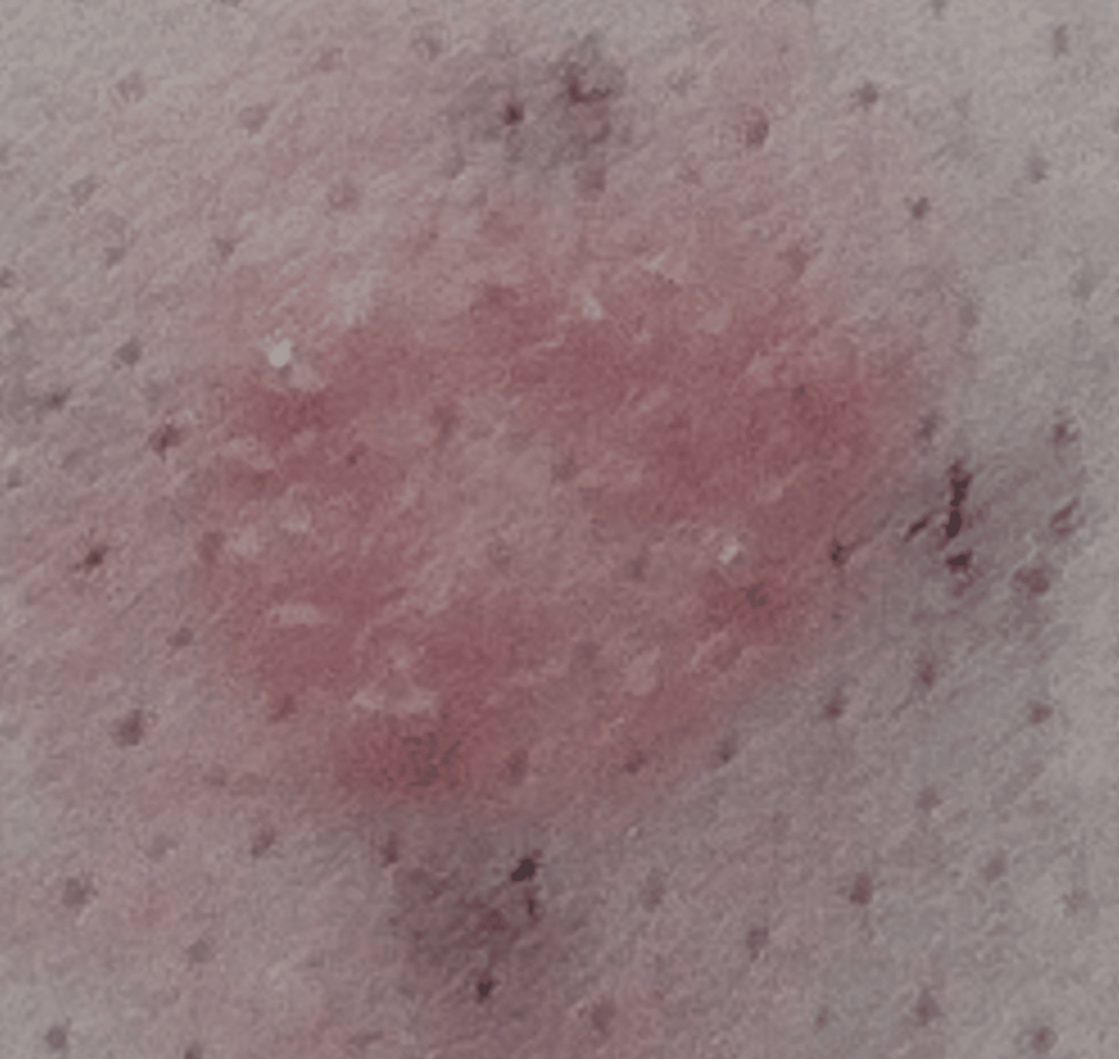
Patch test for nickel. The patient exhibited a cutaneous hypersensitivity to nickel.

The patient also had some allergic reactions to shellfish, including crab, kiwi fruit and pineapple, and certain metals. Before the treatment, patch testing against several metals, including nickel, chromium, stannum, iridium, silver, and cobalt, was performed. Apart from nickel, there was no reaction, but a strong positive result for nickel was observed (Figure 3).

Therefore, we decided to avoid using nickel devices, and a balloon-supported technique was selected for this wide-neck aneurysm.

**Figure 4:**
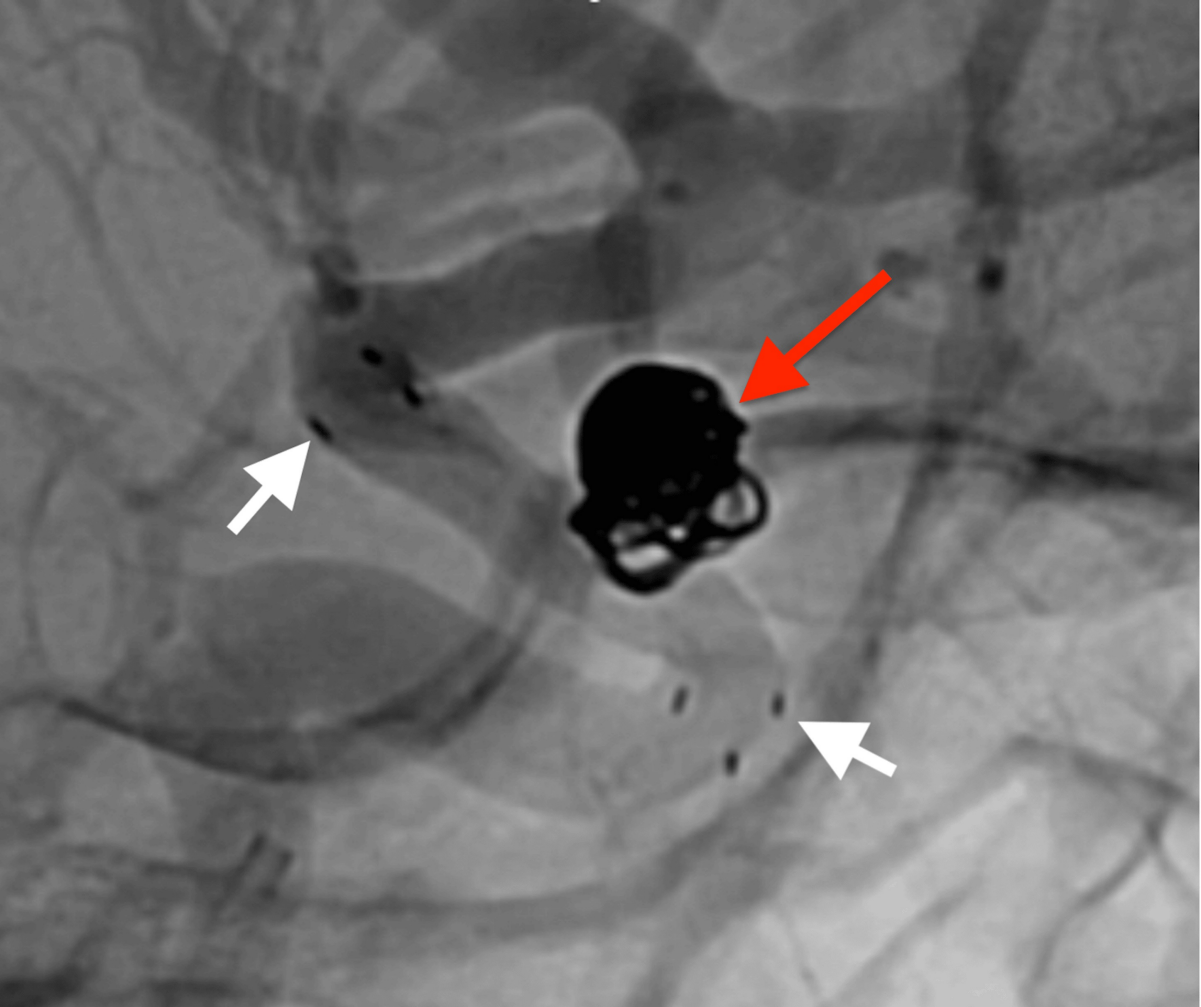
Post-treatment digital angiogram The final digital angiogram after stent-assisted coiling. The distal and proximal tips of the stent are indicated by white arrows, and the embolized aneurysm is indicated by a red arrow. Raymond-Roy occlusion score was class 1.

**Figure 5:**
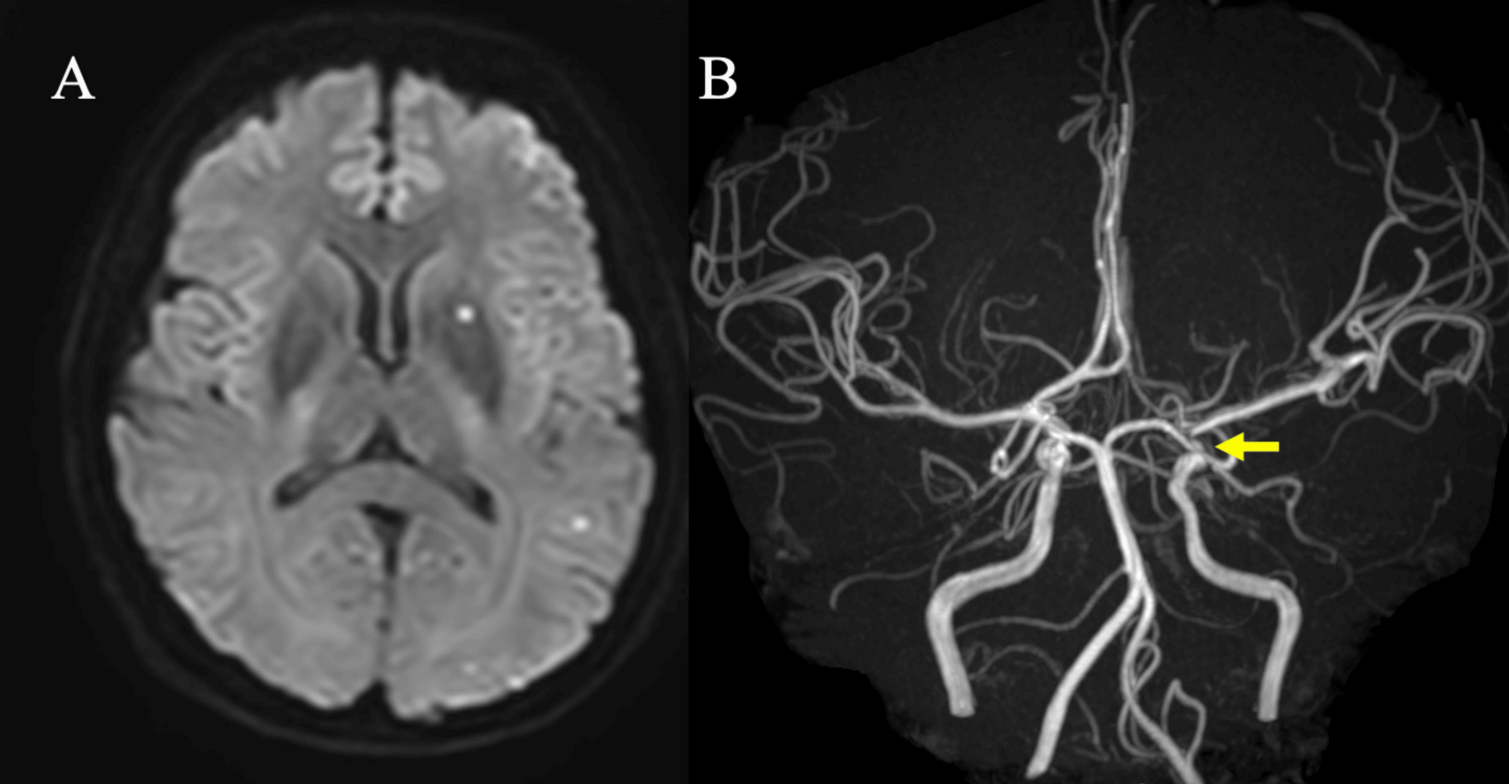
Post-operative MRI images The MRI performed three days post-treatment revealed only several hyperintensity spots on the DWI (A). There were no findings indicative of vessel occlusion due to stent placement, and a left-paraclinoid aneurysm was not identified on the magnetic resonance angiography (B, yellow arrow).

The patient had been taking aspirin 100 mg and prasugrel 3.75 mg for two weeks prior to this treatment. In the evaluation using the LTA method, platelet aggregation was effectively suppressed. Additionally, the VerifyNow™ system (Instrumentation Laboratory, Bedford, MA, USA) was used to assess antiplatelet effects. The aspirin response unit was 403 and the P2Y12 response unit was 100, meaning they adequately inhibited platelet activity. Under local anesthesia, we set the guiding sheath to the left ICA from her right femoral artery. A balloon catheter navigated around the aneurysm. Next, a microcatheter was navigated in the aneurysm with an intermediate catheter. Then, coil embolization was started with a dilated balloon. Good obliteration was achieved until the second coil was inserted. The microcatheter dislodged while inserting the third coil. We then exchanged the microcatheter and continued after reinserting a new one into the aneurysm. However, the sixth coil protruded to the ICA and wobbled. We were concerned that the coils might migrate distally, and thus we deployed a stent (Neuroform Atlas, Stryker Neurovascular, Fremont, CA, USA) to prevent the coils from migrating. After deploying the stent, we got acceptable obliteration (Raymond-Roy occlusion classification 1; Figure 4), and there was no missing branch among the intracranial arteries. After the treatment, we followed up on the patient’s condition at our High Care Unit overnight. As there were no symptoms or abnormal findings on the final angiogram, the patient was followed up without a steroid or an anti-allergic agent. Three days after endovascular treatment, MRI showed only scattered small asymptomatic infarcts, acceptable as post-treatment, and magnetic resonance angiography showed no intracranial arterial occlusion (Figure 5).

**Figure 6:**
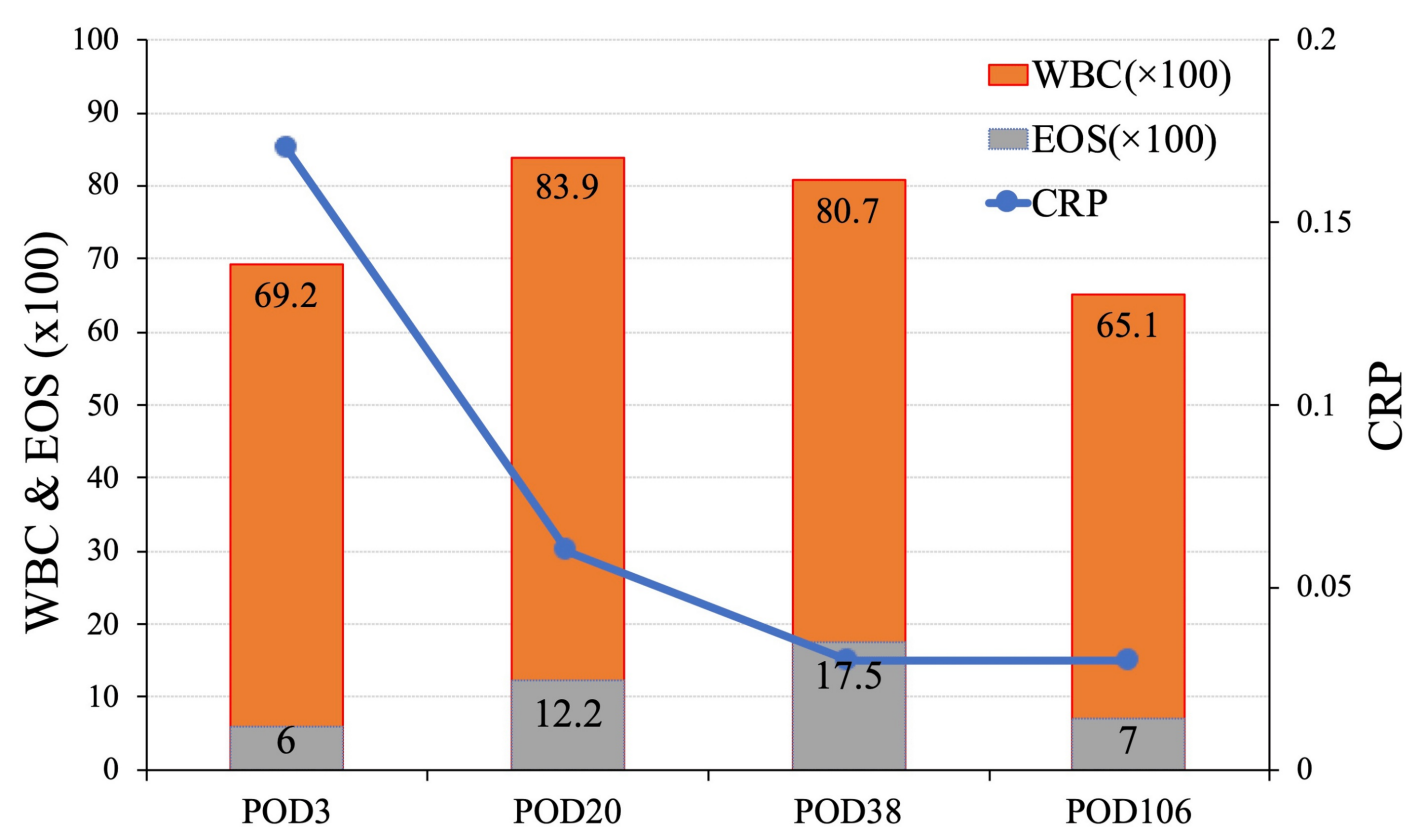
Time course of blood sampling tests On blood examination, transient elevation of WBC and EOS increased transiently, and CRP remained within normal range. WBC, white blood cell; EOS, eosinophil; CRP, C-reactive protein

There were no abnormal findings in the brain suggestive of an allergic reaction. She was discharged with no neurological deficit or any other symptoms. On blood examination, transient elevation of white blood cells (WBC) and eosinophils (EOS) was observed but gradually decreased, and the C-reactive protein (CRP) increased slightly postoperatively but remained within a normal range (Figure 6).

Over a 24-month outpatient follow-up period, the patient's condition had not changed, and there was no edema or recurrence of the aneurysm on MRI.

## Discussion

Due to nickel allergy, balloon-assisted coil embolization of a paraclinoid ICA aneurysm was planned. However, during the procedure, the coil protruded into the ICA, necessitating stent placement. Fortunately, the patient was asymptomatic, with only mildly elevated WBC, EOS, and CRP, and no abnormalities were observed on the MRI over a 24-month follow-up period.

Nickel is a rare metal with stable corrosion resistance in a variety of harsh environments. Pure nickel is difficult to handle, and it is used as stainless steel composed of iron, chromium, and nickel in a wide range of products, from cookware to construction, military, and aerospace applications.

Nickel allergy affects nearly 10-15% of the world's population [1]. Nickel allergy is the most common metal allergy worldwide, is more common in women, and is increasing [2,3]. It has been reported that the incidence rate is 5-20% for women and 2-4% for men [4]. Women have higher risks because of ear piercing, the most common cause of sensitization [5]. Despite this, there are only five reported cases of nickel-related adverse events after intracranial aneurysm endovascular treatment [1]. Such cases are infrequent, and there is a discrepancy between the prevalence of nickel allergy and the incidence rate of allergic reactions after treatment.

Although no postoperative allergy was observed in this case, nickel-induced delayed hypersensitivity reactions have been reported to cause stent restenosis, stent thrombosis, and reversible intraparenchymal changes in allergic patients [6-8]. Diffuse cerebral edema with allergic spasms or focal neurological deficits such as hemiplegia and visual impairment may occur after the implantation of nickel-containing metal in cerebral vessels in patients with nickel allergy, and these symptoms occur one month after treatment [9]. These are treated with high-dose methylprednisolone and improved finally (Table 1) [2,7,9-11].

**Table 1:** Complications related to nickel allergy in stent-assisted coiling (summary of past reports) ICA, internal carotid artery; Lt, left; MCA, middle cerebral artery; Rt, right; VA, vertebral artery

Study	Device	Location	Symptom-related nickel allergy	MRI findings	Treatment
Park et al. [10]	Enterprise (Codman Neurovascular, Raynham, MA, USA)	Lt ICA C3	Right-hand weakness	Multifocal white matter lesion	Steroid
	Enterprise	Rt VA	Abducens nerve palsy, right facial palsy, dysphagia, sensory disturbance	Multifocal white matter lesion	Steroid
Nakagawa et al. [11]	Enterprise	Rt VA	Lt lower motor weakness, dizziness	Multifocal white matter lesion	Steroid
	Neuroform EZ (Stryker Neurovascular, Fremont, CA, USA)	Lt ICA C2	Convulsion	Multifocal white matter lesion	Steroid
Ulus et al. [7]	Enterprise	Lt MCA	Headache, visual disturbance	Multifocal white matter lesion	None

Moreover, there are no reports of dermatological symptoms after stent-assisted coil embolization. Extracranially, several months after implantation of nitinol-containing stents in lower limb arteries or knee prostheses in patients with nickel allergy, there have been reports of marked skin symptoms and swelling, as well as increased CRP, WBC, and EOS [12]. The stent we used had the largest stent strut among VRDs, i.e., the smallest contact area with the vessel. Again, the intracranial stent had a very low absolute amount of metal compared to stents used in other places, which may have prevented a strong allergic reaction. The use of nickel-containing alloys in intracranial devices is essential for endovascular procedures, and most devices are composed of nitinol. This is due to nitinol's exceptional elasticity and shape memory effect. Nickel is one of nitinol's significant components, accounting for 55%, which supports its shape memory with intracranial VRDs [2].

Generally, paraclinoid aneurysm is treated with surgical clipping or endovascular therapy. An alternative treatment for patients with nickel allergy, where the use of stents or other devices made of various alloys containing nickel other than platinum coils is contemplated, is craniotomy clipping. Clips and bone fixation plates used in clipping procedures are made of titanium and, therefore, do not contain nickel. When treated by surgical clipping for aneurysms with a pterional approach, including IC paraclinoid aneurysms, ophthalmic arteries branching ectopically from the MMA should be noted, as they account for 3.5% of cases [13]. Surgical clipping has a high risk of intraoperative injury to the MMA, and there have been reports of blindness [14]. In this situation, endovascular therapy is often favorable. In contrast, thromboembolic complications of endovascular treatment of paraclinoid aneurysms have been reported to range from 2.8% to 5.4%, with no postoperative neurological symptoms [15].

Our case has a limitation. According to the previous report, some reactions due to nickel allergy happened one month after endovascular procedures, but there is also a report that it came 12 months later [2]. In our case, follow-up MRI two years post-procedure demonstrated no abnormal findings, and the patient remained asymptomatic. However, the potential for a delayed allergic reaction exists, and thus we need to observe the patient's condition continuously to check whether the patient has some complications.

## Conclusions

We reported the case of unavoidable stent-assisted coiling for a patient who had a nickel allergy. There were no complications, including MRI, except for transient elevation of EOS. Furthermore, the patient presented with an ophthalmic artery branching off the middle meningeal artery, which could have resulted in a reduction in ophthalmic artery blood flow and subsequent blindness following craniotomy. While severe nickel allergic complications may not be confirmed in every nickel allergy patient, it is of the utmost importance that careful observation be conducted on a consistent long-term basis in the future.
